# Nutritional status and cancer survival among rural Chinese women: biological mechanisms, health disparities, and translational opportunities

**DOI:** 10.3389/fpubh.2026.1887699

**Published:** 2026-06-12

**Authors:** Kelun Zhang, Bing Wang, Yuqin Ji, Huiming Jiang

**Affiliations:** 1 College of Economics and Management, Jilin Agricultural University, Changchun, China; 2 Institute of Special Environmental Medicine, Nantong University, Nantong, China; 3 School of Economics and Management, Changzhou Institute of Technology, Changzhou, China

**Keywords:** cancer nutrition, gut microbiome, health disparities, metabolic signaling, nutritional status, rural China, women’s cancers

## Abstract

Cancer remains a major cause of premature mortality among women worldwide, and its burden is particularly pronounced in rural China, where delayed diagnosis, uneven access to oncology services, and nutritional vulnerability may jointly affect survival. This narrative review synthesizes mechanistic, clinical, and population-level evidence on the relationship between nutritional status and cancer survival among rural Chinese women, with a focus on breast, cervical, gastric, and colorectal cancers. It first outlines the epidemiological profile of major female cancers in rural China and summarizes persistent rural–urban disparities in cancer incidence, stage at diagnosis, treatment access, and survival. It then examines nutrition-related challenges in rural settings, including dietary transition, micronutrient insufficiency, metabolic vulnerability, food insecurity, limited dietary diversity, and the increasing availability of energy-dense ultra-processed foods. The biological pathways linking nutritional status to cancer progression, treatment tolerance, and survivorship are discussed across four interconnected domains: insulin–IGF-1 and AMPK–mTOR signaling, adiposity-related inflammation and tumor microenvironment remodeling, gut microbiome–diet–metabolite interactions affecting estrogen metabolism, and micronutrient-dependent epigenetic regulation. Available clinical and epidemiological evidence on dietary patterns, nutritional biomarkers, and cancer prognosis in Chinese women is reviewed, with attention to methodological limitations and the shortage of rural-specific longitudinal data. The review further considers how food insecurity, low nutrition literacy, weak integration of oncology and nutrition services, and structural inequities in rural health systems may amplify survival disparities. Finally, translational opportunities are discussed, including community-based nutritional screening, integration of nutrition assessment into county-level oncology care, digital health tools, and scalable dietary counseling models adapted to rural contexts. Overall, this review highlights the need for prospective cohort studies with repeated nutritional biomarker assessments, mechanistic validation in rural populations, and equity-oriented policy strategies to improve cancer survivorship among rural Chinese women.

## 1 Introduction

Cancer has become one of the leading causes of premature mortality in China, and its burden continues to shape public health priorities for women across both urban and rural settings (1). Recent national cancer statistics show that lung, breast, thyroid, colorectal, and cervical cancers are among the most frequently diagnosed malignancies in Chinese women, while gastrointestinal cancers remain clinically important because of their high mortality and uneven regional distribution (2). Although cancer survival in China has improved over recent decades, population-based registry evidence still shows clear disparities by region, healthcare access, and cancer type (3).

These inequalities are particularly relevant for rural women. Compared with women living in urban areas, rural women are more likely to encounter delayed diagnosis, limited screening access, geographic barriers to tertiary oncology services, and fragmented follow-up after treatment (4). Such disadvantages may affect not only the timing of diagnosis but also treatment continuity, nutritional management, rehabilitation, and long-term survivorship. For cancers such as breast, cervical, gastric, and colorectal cancer, rural disadvantage should therefore be understood as a cumulative process that extends across the full cancer care trajectory rather than a single barrier at diagnosis.

Rural women represent a distinct review population because gendered caregiving roles, agricultural labor, lower mobility, uneven nutrition literacy, and reliance on county-level or referral-based care can intersect with cancer treatment demands. Rural China is also heterogeneous: food access, screening coverage, income, ethnicity, migration patterns, and local dietary traditions differ across provinces and villages. Therefore, the review uses rural–urban comparisons as a framework for identifying plausible inequities, not as a claim that all rural regions or patients share the same risk profile.

Nutritional status is increasingly recognized as a clinically modifiable factor in oncology care. International cancer nutrition guidelines emphasize that malnutrition and metabolic derangements should be identified and managed early because they may worsen treatment tolerance, increase complications, reduce functional status, and compromise quality of life (5). Clinical evidence further suggests that poor nutritional status, low albumin, reduced muscle reserve, and malnutrition-based prognostic scores are associated with unfavorable outcomes in several cancer populations (6). Conversely, healthier dietary patterns characterized by higher intake of vegetables, fruits, whole grains, soy products, fish, and other nutrient-dense foods have been associated with better outcomes in female cancer cohorts (7).

For rural Chinese women, nutrition is not only an individual behavioral issue but also a socially patterned determinant of survival. Rural China has experienced a rapid and uneven nutrition transition, in which traditional diets based on grains, legumes, vegetables, and minimally processed foods have increasingly coexisted with refined carbohydrates, processed snacks, animal-source foods, and ultra-processed products (8). This transition has created a double burden in which micronutrient insufficiency, low dietary diversity, overweight, central adiposity, and metabolic dysregulation may appear within the same communities and sometimes within the same individuals (9). These challenges may be intensified by food insecurity, seasonal variation in fresh produce availability, limited nutrition literacy, and weak integration of nutrition services into oncology care (10).

**Figure 1:**
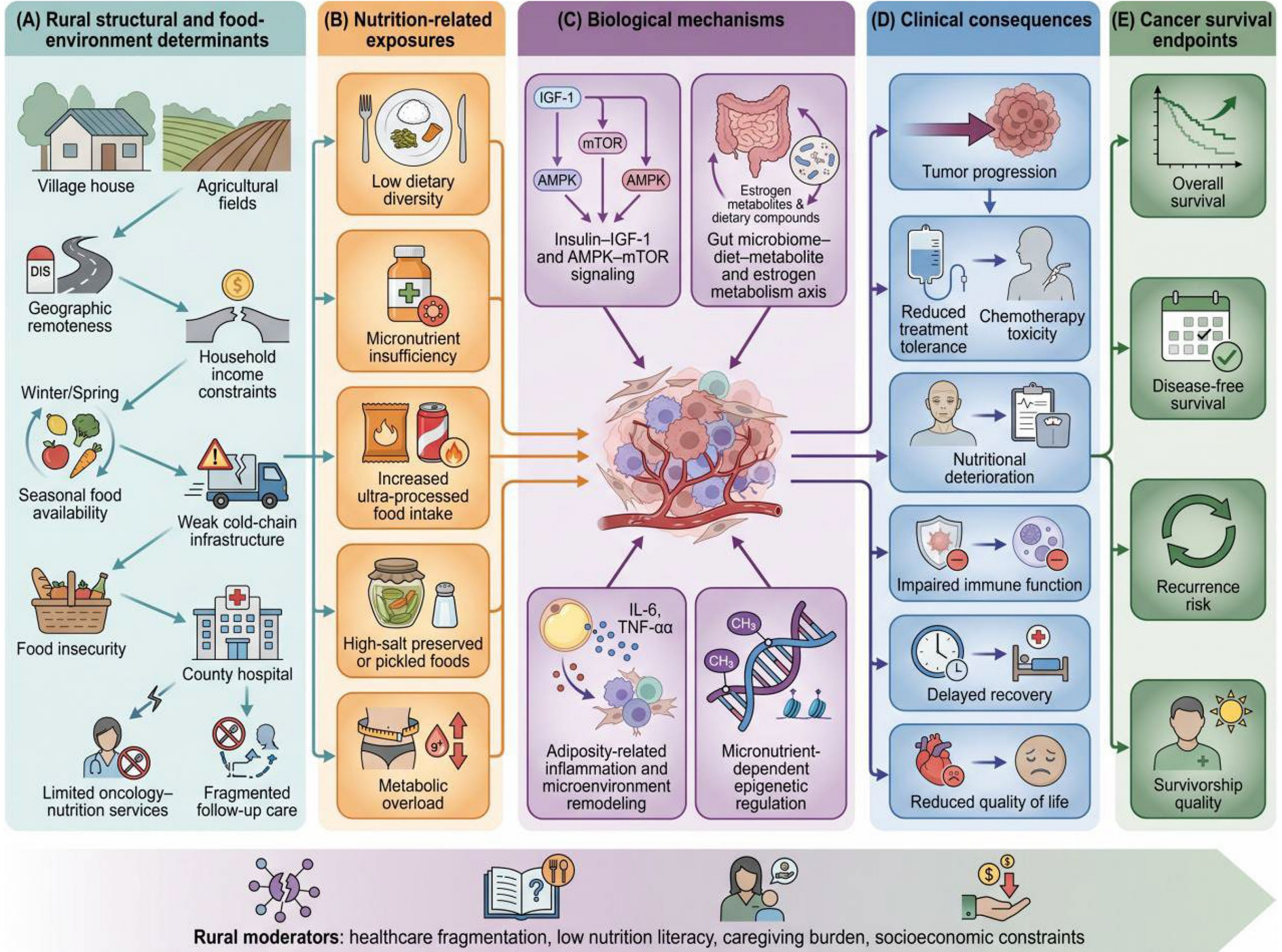
Conceptual framework linking rural nutritional vulnerability to cancer survival among women in China. **(A)** Rural structural and food-environment determinants include household income constraints, geographic remoteness, seasonal fresh food availability, weak cold-chain infrastructure, food insecurity, limited oncology–nutrition integration, and fragmented follow-up care. **(B)** These determinants shape nutrition-related exposures, including low dietary diversity, micronutrient insufficiency, increased consumption of ultra-processed foods, high-salt preserved foods, and metabolic overload. **(C)** Nutrition-related exposures influence cancer biology through interconnected mechanisms, including insulin–IGF-1 and AMPK–mTOR signaling, adiposity-related inflammation, tumor microenvironment remodeling, gut microbiome–diet–metabolite interactions, estrogen metabolism, and micronutrient-dependent epigenetic regulation. **(D)** These mechanisms may affect clinical outcomes through tumor progression, reduced treatment tolerance, nutritional deterioration, impaired immune function, delayed recovery, and reduced quality of life. **(E)** The final pathway links these clinical processes to cancer survival endpoints, including overall survival, disease-free survival, recurrence risk, and survivorship quality. Cross-cutting rural moderators, including healthcare fragmentation, low nutrition literacy, caregiving burden, and socioeconomic constraints, influence each stage of the pathway.

To clarify how these issues are connected, this review conceptualizes rural nutritional vulnerability as a pathway linking household food environments, biological mechanisms, treatment tolerance, and survival outcomes. As shown in Figure 1, nutritional status is not treated here as a single dietary exposure, but as an intersecting determinant shaped by rural food systems, healthcare access, socioeconomic constraints, and cancer-related metabolic demands.

Against this background, this narrative review synthesizes existing evidence across three domains: the epidemiological and nutritional relevance of major female cancers in rural China; the biological pathways through which nutritional status may influence tumor biology, immune function, treatment response, and survivorship; and the clinical, public health, and policy implications for improving nutrition-related cancer care in rural settings. Rather than treating nutrition solely as an individual lifestyle factor, this review positions nutritional status as a biologically active and socially patterned determinant of cancer survival. The aim is to provide a framework that connects mechanistic evidence with rural health disparities and identifies priorities for future research, clinical translation, and equity-oriented intervention.

## 2 Epidemiological mapping of female cancer in rural China

The cancer burden among Chinese women has changed substantially over the past three decades. National cancer registry estimates indicate that breast cancer is now one of the most common malignancies in women, while lung, colorectal, thyroid, cervical, gastric, and other digestive cancers continue to contribute substantially to the overall burden (11). However, the cancer profile of rural women differs from national averages in several important ways. This review focuses on breast and cervical cancer because they combine high female cancer burden, clear survivorship relevance, and specific links to nutrition, screening access, or rural food environments.

Gastrointestinal cancers, including gastric and esophageal cancer, remain disproportionately relevant in some rural and north-central regions, where dietary patterns have historically included higher consumption of salt-preserved foods, pickled vegetables, and other preserved products (12). These exposures are important because preserved and pickled foods have been associated with increased gastric cancer risk in epidemiological studies (13). Cervical cancer also remains a major concern in rural settings, where uneven screening coverage and persistent high-risk human papillomavirus infection may increase the likelihood of delayed detection (14).

Rural women are also more likely to experience later diagnosis and less continuous access to standardized treatment pathways. Earlier multicenter evidence on breast cancer in China showed regional and institutional differences in clinical presentation and care patterns, suggesting that access to timely diagnosis and specialist treatment remains uneven (15). Population-based survival analyses further indicate that survival improvement has not eliminated disparities across cancer types and regions, which is highly relevant for rural women who depend on county-level or referral-based cancer care systems (16).

These disparities arise from interacting structural, behavioral, clinical, and biological determinants. Existing literature has documented delayed diagnosis and unequal access to treatment, but the contribution of nutritional status to rural cancer outcomes remains less clearly defined. This gap is important because nutritional vulnerability may influence cancer survival through impaired treatment tolerance, reduced immune competence, greater susceptibility to treatment-related toxicity, poor postoperative recovery, and altered tumor-promoting metabolic environments. Understanding the nutritional dimension of rural cancer disparities therefore requires moving beyond general descriptions of healthcare access and examining how diet, body composition, micronutrient status, and food environments may shape cancer trajectories among rural women.

## 3 Nutritional challenges in rural settings in China

Rural nutritional vulnerability is produced by overlapping dietary, socioeconomic, geographic, and healthcare-system factors. These factors do not operate independently. Instead, they interact across the cancer care trajectory, from baseline nutritional reserves before diagnosis to treatment tolerance, recovery, follow-up, and long-term survivorship. Table 1 summarizes the major nutrition-related challenges in rural settings and their potential implications for cancer outcomes among women.

**Table 1:** Nutrition-related challenges in rural settings and their potential implications for cancer survival among women in China.

Rural nutrition-related challenge	Main rural expression	Potential biological or clinical implication	Relevance to cancer survival
Dietary transition	Increasing availability of refined staples and ultra-processed foods through rural retail networks, including refined white rice or wheat-flour staples, instant noodles, packaged biscuits or cakes, processed meats, and sugar-sweetened drinks	Higher risk of excess energy intake, central adiposity, insulin resistance, and metabolic dysregulation	May contribute to tumor-promoting metabolic environments and reduced treatment tolerance
Low dietary diversity	Narrow range of nutrient-dense foods, especially fresh vegetables and fruits, legumes or soy foods, coarse grains, and high-quality protein; this refers mainly to dietary variety rather than total household food adequacy	Micronutrient insufficiency, reduced antioxidant intake, impaired immune function, and poorer functional reserve	May increase vulnerability to treatment-related toxicity, postoperative complications, and functional decline
Dependence on preserved and pickled foods	Continued use of salt-preserved vegetables, pickled foods, cured meats, and high-salt dietary patterns in some rural regions	Increased exposure to salt, N-nitroso compounds, and gastrointestinal mucosal irritation	Strongest relevance is for gastrointestinal cancer risk; post-diagnosis survival implications are less direct and may relate mainly to overall diet quality and baseline nutritional status
Food insecurity	Economic, geographic, or treatment-related difficulty obtaining sufficient, safe, affordable, and symptom-tolerant foods; this refers to household food access rather than dietary variety alone	Inadequate protein-energy intake, low dietary quality, and difficulty maintaining nutritional status during treatment	May accelerate treatment-associated weight loss, cachexia risk, and poor recovery
Seasonal food availability	Periodic fluctuation in fresh produce and protein supply caused by growing seasons, weather, market distance, income instability, and weak cold-chain infrastructure; this refers to temporal instability rather than chronic access alone	Inconsistent nutrient intake and poor adherence to dietary recommendations	May reduce feasibility of sustained dietary support during chemotherapy, radiotherapy, and survivorship
Limited nutrition literacy	Low awareness of cancer-related nutritional needs and persistence of dietary misconceptions during treatment	Restrictive eating, avoidance of protein-rich foods, delayed nutritional support, or reliance on unverified dietary practices	May worsen malnutrition, reduce treatment adherence, and impair recovery
Weak oncology–nutrition integration	Limited availability of dietitians, nutritional screening, and structured dietary counseling in county- and township-level cancer care	Delayed identification of malnutrition and lack of individualized nutrition intervention	May contribute to preventable treatment interruption, poorer tolerance, and survival disadvantage
Caregiving and labor burden among rural women	Left-behind women, older women, and women in agricultural households may carry both household and productive labor responsibilities	Reduced time and resources for self-care, follow-up visits, and nutritional management	May amplify rural disadvantage across diagnosis, treatment, and long-term survivorship

### 3.1 Dietary transition and the nutritional double burden

Rural China is experiencing a complex dietary transition rather than a simple shift from undernutrition to overnutrition. Longitudinal evidence from the China Health and Nutrition Survey has shown substantial changes in dietary structure over time, including increased intake of edible oils, animal-source foods, refined products, and processed foods, alongside declining reliance on traditional plant-based dietary patterns in many communities (17). This shift is relevant to cancer survivorship because diet quality, body composition, metabolic status, and inflammatory burden may influence treatment tolerance and recovery.

Although extreme food insufficiency has declined, many rural households continue to face limited dietary diversity, seasonal instability in fresh food availability, and uneven access to nutrient-rich foods. These challenges are especially relevant for older women, left-behind women whose spouses have migrated for work, and women living in western or minority regions where household labor demands, geographic remoteness, and economic constraints may restrict dietary choice (18). For women with cancer, such constraints may reduce baseline nutritional reserve before surgery, chemotherapy, radiotherapy, or long-term endocrine treatment.

At the same time, the rural food environment has changed rapidly. Processed snacks, refined carbohydrates, sugar-sweetened beverages, and other energy-dense products have become increasingly accessible through rural retail networks, while traditional dietary patterns based on grains, legumes, vegetables, and minimally processed foods have weakened in some communities (19). This transition may increase the coexistence of micronutrient insufficiency, excess energy intake, central adiposity, and metabolic dysregulation. For women undergoing cancer treatment, this double burden may reduce treatment tolerance, increase functional decline, and complicate long-term survivorship.

### 3.2 Food insecurity and inequitable food environments

Food insecurity in rural China should be understood not only as insufficient calorie intake, but also as restricted access to diverse, safe, affordable, and nutritionally adequate foods. Even where severe hunger has become less common, qualitative food insecurity may persist through low dietary diversity, limited fresh produce availability, dependence on preserved or pickled foods, and reduced access to high-quality protein sources (20). These constraints are particularly important for women with cancer, whose nutritional needs may increase during treatment while appetite, digestion, and food tolerance often decline.

Inequitable food environments may further limit adherence to dietary recommendations during cancer care. Seasonal fluctuations in fruit and vegetable supply, weak cold-chain infrastructure, household income constraints, and the growing availability of ultra-processed foods can make evidence-based dietary advice difficult to implement in daily life (21). For rural women, these barriers are often compounded by caregiving responsibilities, agricultural labor, limited nutrition literacy, and fragmented follow-up after treatment at higher-level hospitals. As a result, nutritional decline during cancer treatment may not be detected early, and practical dietary support may remain unavailable at the point where it is most needed. In practical policy terms, local agricultural cooperatives, village markets, and community food-supply systems could help stabilize access to fresh produce, legumes, soy products, eggs, and other affordable protein sources during treatment and survivorship.

These food-system and healthcare-system constraints provide a plausible pathway through which rural disadvantage may contribute to poorer treatment tolerance, slower recovery, and less favorable survival outcomes. This pathway is especially important because nutrition-related risk is not fixed at diagnosis; it may worsen during treatment, persist through survivorship, and interact with recurrence risk, functional decline, and quality of life. Rural cancer nutrition should therefore be addressed as a longitudinal care issue rather than as a brief dietary recommendation delivered at a single clinical encounter.

## 4 Biological mechanisms linking nutritional status to cancer outcomes in women

**Figure 2:**
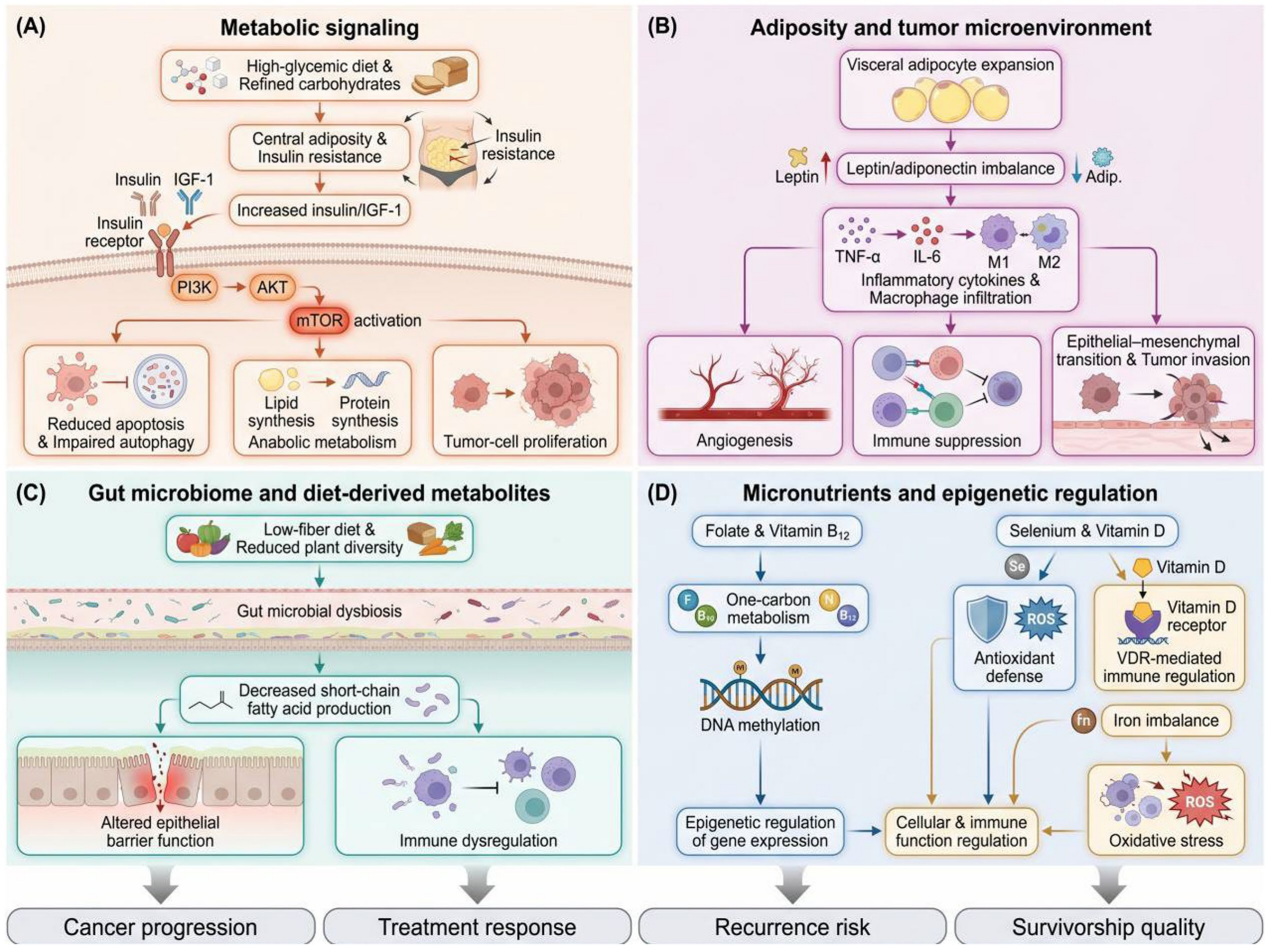
Multi-pathway biological mechanisms linking nutritional status to cancer outcomes in women. **(A)** Metabolic signaling pathway showing how positive energy balance, high-glycemic diets, insulin resistance, and central adiposity may activate insulin–IGF-1 and PI3K–AKT–mTOR signaling, thereby promoting tumor-cell proliferation, anabolic metabolism, reduced apoptosis, and impaired autophagy. **(B)** Adiposity and tumor microenvironment pathway showing how visceral adiposity and low muscle reserve may contribute to adipokine imbalance, chronic inflammation, angiogenesis, immune suppression, epithelial–mesenchymal transition, and tumor invasion. **(C)** Gut microbiome pathway showing how low-fiber and low-diversity diets may reduce microbial diversity, decrease short-chain fatty acid production, alter epithelial barrier regulation, and disturb immune homeostasis. **(D)** Micronutrient and epigenetic pathway showing how folate, vitamin B12, selenium, vitamin D, and iron status may influence DNA methylation, oxidative stress, immune function, host resilience, and treatment tolerance. These four pathways converge on cancer progression, treatment response, recurrence risk, and survivorship quality.

Nutritional status may influence cancer outcomes through several interconnected biological pathways. For rural women, these mechanisms are particularly relevant because dietary transition, low dietary diversity, micronutrient insufficiency, food insecurity, excess adiposity, and treatment-related nutritional decline may coexist across the cancer care trajectory. Rather than acting as isolated exposures, these factors may shape the metabolic, inflammatory, microbial, and epigenetic conditions in which tumors progress and treatments are tolerated. Figure 2 summarizes the major biological pathways through which nutritional vulnerability may affect tumor progression, treatment response, recurrence risk, and survivorship.

Table 2 provides a mechanistic bridge between the rural nutrition-related challenges described above and the clinical evidence discussed in the following section. It links each major nutritional vulnerability with plausible biological pathways and cancer-relevant outcomes.

**Table 2:** Mechanistic pathways linking nutritional vulnerability to cancer outcomes among rural women.

Nutritional exposure or vulnerability	Main biological pathway	Cancer-relevant mechanism	Potential clinical implication
Positive energy balance, high-glycemic diets, and refined carbohydrate intake	Insulin–IGF-1 and PI3K–AKT–mTOR signaling	Increased insulin and IGF-1 signaling may promote cellular proliferation, anabolic metabolism, reduced apoptosis, and altered stress-response regulation	May create a tumor-promoting metabolic environment and reduce metabolic resilience during treatment
Central adiposity with low muscle reserve	Adipokine imbalance and chronic low-grade inflammation	Visceral adiposity may increase inflammatory signaling, alter leptin/adiponectin balance, promote immune suppression, and remodel the tumor microenvironment	May worsen treatment tolerance, functional recovery, recurrence risk, and survivorship quality
Low-fiber and low-diversity diets	Gut microbiome disruption and reduced short-chain fatty acid production	Reduced microbial diversity and lower butyrate production may weaken epithelial barrier regulation, anti-inflammatory signaling, and immune homeostasis	May reduce host resilience and could affect treatment response; microbiome-targeted interventions require validation before routine clinical use
Transitional high-fat and low-fiber dietary patterns	Gut microbiome–estrogen metabolism axis	Altered microbial β-glucuronidase activity may increase enterohepatic estrogen recirculation and systemic free estrogen exposure	May influence hormone-sensitive tumor biology, especially in breast and endometrial cancer contexts
Folate and vitamin B12 insufficiency	One-carbon metabolism and DNA methylation	Insufficient methyl-donor availability may alter DNA methylation, nucleotide synthesis, genomic stability, and cellular repair processes	May contribute to epigenetic dysregulation and impaired cellular homeostasis
Selenium insufficiency	Redox regulation and antioxidant defense	Reduced selenoprotein activity may weaken antioxidant defense and increase vulnerability to oxidative DNA damage	May increase oxidative stress burden; supplementation should be deficiency-based because benefits remain uncertain and safety margins may be narrow
Vitamin D insufficiency	Vitamin D receptor-mediated immune and transcriptional regulation	Low vitamin D status may affect immune modulation, cell differentiation, inflammatory balance, and host recovery capacity	May influence treatment resilience and functional recovery, but causal effects require further validation
Iron deficiency, anemia, or iron imbalance	Oxygen transport, immune function, and oxidative stress regulation	Iron deficiency may impair immune competence and treatment tolerance, while excess iron may contribute to oxidative damage through redox reactions	May affect chemotherapy tolerance, fatigue, recovery, and systemic vulnerability
Treatment-related anorexia, nausea, mucositis, diarrhea, or malabsorption	Protein-energy depletion and systemic inflammation	Reduced intake and absorption may lead to weight loss, sarcopenia, immune weakness, and treatment interruption	May worsen toxicity, delay recovery, reduce quality of life, and compromise survival outcomes

### 4.1 Metabolic signaling: insulin–IGF-1 and AMPK–mTOR pathways

A sustained positive energy balance, high-glycemic dietary intake, insulin resistance, and central adiposity may activate metabolic signaling pathways that favor tumor growth. Insulin and insulin-like growth factor 1 may stimulate downstream PI3K–AKT–mTOR signaling, thereby supporting cellular proliferation, anabolic metabolism, reduced apoptosis, and altered stress-response regulation (22). This pathway is relevant to women’s cancers because metabolic dysregulation may interact with hormone-sensitive tumor biology, particularly in postmenopausal women and in patients with central adiposity.

The counter-regulatory AMPK pathway provides a complementary mechanism. AMPK activation can inhibit mTOR signaling, support cellular energy homeostasis, and promote adaptive responses to metabolic stress (23). In cancer care, this does not justify unsupervised calorie restriction or extreme dietary practices, especially in patients at risk of malnutrition. Instead, it highlights why metabolic status should be considered part of the treatment context. For rural women undergoing dietary transition, increased intake of refined carbohydrates and energy-dense foods may contribute to insulin resistance and metabolic inflammation, while inadequate intake during treatment may simultaneously reduce nutritional reserve. This dual risk makes metabolic balance, rather than simple caloric reduction, the more clinically relevant target.

### 4.2 Adiposity, inflammation, and tumor microenvironment remodeling

Excess adiposity, especially visceral adiposity, may reshape the tumor microenvironment through chronic low-grade inflammation, adipokine imbalance, and immune dysregulation. Obesity-related inflammation is associated with increased inflammatory mediators, altered macrophage activity, angiogenesis, immune suppression, epithelial–mesenchymal transition, and greater invasive potential (24). This mechanism is important in rural China because nutritional vulnerability does not only mean undernutrition. It may also involve overweight, central obesity, low muscle reserve, and poor diet quality within the same patient population.

Adipose tissue also has endocrine activity that is particularly relevant to women’s cancers. In postmenopausal women, adipose tissue may contribute to estrogen production through aromatase activity, thereby plausibly supporting hormone-sensitive tumor growth and recurrence-related pathways (25). These mechanisms suggest that nutritional assessment in rural women should not be limited to body weight alone. Body composition, metabolic status, inflammatory burden, and functional reserve are all clinically meaningful because they may influence tumor biology, treatment tolerance, and survivorship quality. In low-resource settings, feasible indicators include BMI, waist circumference, recent unintentional weight change, reduced intake, hand-grip strength when a dynamometer is available, gait speed, chair-stand performance, or a simple functional assessment of daily activity and fatigue.

### 4.3 Gut microbiome, diet-derived metabolites, and estrogen metabolism

The gut microbiome is an important mediator between diet, host metabolism, immune function, and cancer biology. Dietary composition can alter microbial community structure and influence the production of bioactive metabolites, including short-chain fatty acids, secondary bile acids, and tryptophan-derived metabolites that may affect inflammation, epithelial integrity, immune regulation, and therapeutic response (26). Diets low in fiber and plant diversity may reduce short-chain fatty acid production, particularly butyrate, which is linked to anti-inflammatory and epithelial regulatory functions.

**Figure 3:**
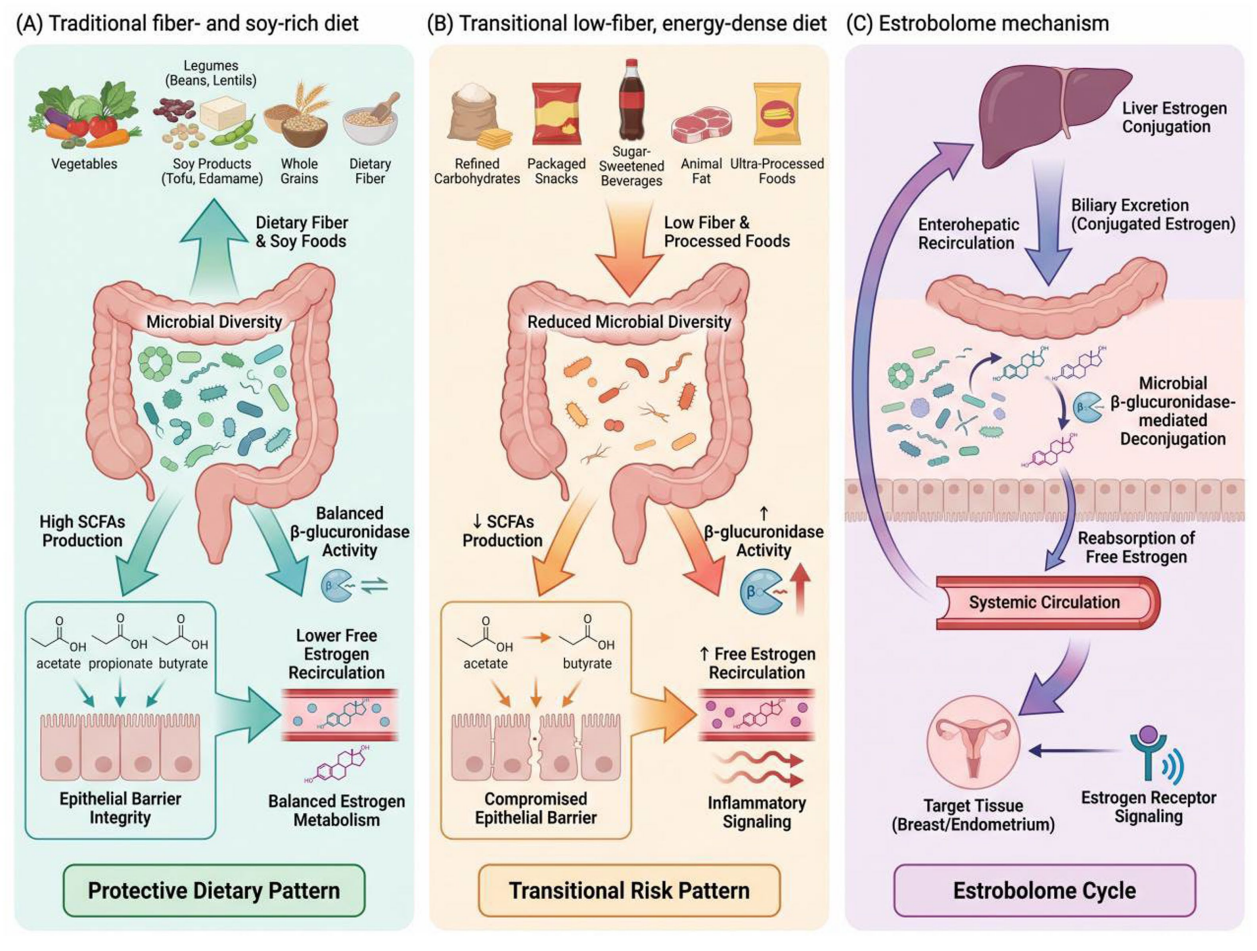
Diet–microbiome–estrogen metabolism axis in women’s cancer biology. **(A)** A traditional rural dietary pattern rich in fiber, legumes, soy products, whole grains, and vegetables may support microbial diversity, short-chain fatty acid production, epithelial barrier integrity, and balanced estrogen metabolism. **(B)** A transitional dietary pattern characterized by reduced fiber intake, refined carbohydrates, animal-source foods, and ultra-processed products may reduce microbial diversity, decrease short-chain fatty acid production, increase *β*-glucuronidase activity, and promote greater enterohepatic recirculation of free estrogens. **(C)** The estrobolome mechanism involves hepatic estrogen conjugation, biliary excretion into the intestine, microbial β-glucuronidase-mediated deconjugation, reabsorption of free estrogens, and estrogen receptor activation in hormone-sensitive tissues. This axis provides a plausible biological link between dietary transition, gut microbial metabolism, estrogen exposure, and women’s cancer biology.

For women’s cancers, the microbiome–estrogen axis deserves particular attention. The estrobolome refers to gut microbial genes involved in estrogen metabolism; *β*-glucuronidase-producing bacteria can deconjugate estrogens in the intestinal lumen, allowing free estrogens to be reabsorbed into circulation (27). This pathway provides a plausible mechanism through which low-fiber, high-fat, and low-diversity diets may influence systemic estrogen exposure and hormone-sensitive cancer biology. Figure 3 illustrates this diet–microbiome–estrogen metabolism axis by contrasting a traditional fiber- and soy-rich dietary pattern with a transitional diet characterized by lower fiber intake, greater refined carbohydrate intake, and increased animal-source or processed foods. At present, these microbiome mechanisms should not be treated as direct clinical instructions on their own; probiotic, prebiotic, or other microbiome-targeted interventions require further validation in cancer-specific and rural populations before routine recommendation.

### 4.4 Micronutrient insufficiency, oxidative stress, and epigenetic dysregulation

Micronutrient insufficiency may influence cancer outcomes through DNA repair, methylation, oxidative stress, immune competence, and treatment tolerance. Folate and vitamin B12 are central to one-carbon metabolism, which supplies methyl groups for DNA methylation and supports nucleotide synthesis and genomic stability (28). Inadequate folate or vitamin B12 status may therefore affect epigenetic regulation and cellular repair capacity, although the direction of effect can vary by dose, timing, tissue context, and cancer type.

Selenium is relevant because it contributes to selenoprotein-mediated antioxidant defense and redox regulation. Selenium insufficiency may weaken protection against oxidative DNA damage, particularly in regions where soil selenium availability is low (29). Vitamin D status is also clinically important because vitamin D receptor signaling is involved in immune modulation, cell differentiation, inflammation, and host resilience (30). These micronutrient pathways should not be interpreted as support for indiscriminate supplementation. A more clinically defensible approach is to identify and correct meaningful deficiencies, especially in patients with poor dietary intake, treatment-related anorexia, anemia, weight loss, or functional decline.

## 5 Clinical and epidemiological evidence

### 5.1 Dietary patterns and cancer survivorship among Chinese women

Clinical and epidemiological evidence suggests that diet quality may influence cancer prognosis, but the evidence base remains uneven. Post-diagnosis soy food intake has been examined in large breast cancer survivor cohorts, and pooled evidence from Chinese and US women suggests that soy consumption after diagnosis is not associated with poorer outcomes and may be linked to lower recurrence risk in some groups (31). This finding is relevant because soy foods have historically been part of several Chinese dietary patterns, although intake varies by region, age, income, and degree of dietary transition.

Broader dietary-pattern evidence also supports the idea that survivorship nutrition should focus on overall diet quality rather than single foods alone. Diets with greater intake of plant foods, whole grains, legumes, fish, and other nutrient-dense items may support metabolic health and functional recovery, whereas high intake of refined carbohydrates, processed meats, and energy-dense foods may worsen metabolic risk (32). However, most available survivorship data come from urban or mixed urban–rural cohorts. Direct evidence from rural Chinese women with cancer remains limited.

This gap matters because rural dietary exposures may differ meaningfully from urban patterns. In some rural regions, preserved foods, pickled vegetables, high-salt dietary practices, limited fresh produce diversity, and uneven protein access may shape baseline nutritional status before diagnosis. During cancer treatment, these exposures interact with anorexia, nausea, mucositis, diarrhea, surgical malabsorption, fatigue, and financial constraints. Therefore, the key research question is not simply whether a specific food protects against or increases cancer risk, but whether the rural food environment supports treatment tolerance, recovery, and long-term survivorship. For preserved vegetables, pickled foods, and high-salt dietary practices, the strongest available evidence relates to gastrointestinal cancer risk (13, 33); evidence for post-diagnosis treatment tolerance, recurrence, or survival remains less direct and should be interpreted cautiously.

### 5.2 Nutritional biomarkers and prognostic significance

Nutritional biomarkers and structured nutritional assessment tools provide a more clinically actionable way to evaluate risk than dietary recall alone. Global Leadership Initiative on Malnutrition (GLIM)-defined malnutrition has been associated with poorer overall survival and greater postoperative risk among cancer patients, supporting the prognostic value of systematic malnutrition assessment (6). This is particularly relevant for rural women because unrecognized weight loss, low muscle reserve, anemia, reduced intake, and micronutrient insufficiency may already be present before treatment begins.

Composite nutritional and inflammatory indices, including albumin-based scores, prognostic nutritional indices, hemoglobin-related measures, lymphocyte-based indices, and body composition measures, can help identify patients at higher risk of poor treatment tolerance and postoperative complications (34, 35). These markers are not perfect and should not replace clinical assessment. Still, they can provide a practical entry point for county-level hospitals where advanced dietetic services, imaging-based body composition analysis, or repeated metabolomic testing may not be feasible. Low-cost first-line indicators include BMI, recent unintentional weight loss, reduced intake, serum albumin, hemoglobin, total lymphocyte count, and symptom burden; where feasible, waist circumference, hand-grip strength, gait speed, or chair-stand performance can be added.

Emerging metabolomic and microbiome approaches may eventually improve nutritional phenotyping. Plasma and urine metabolomic profiles can capture amino acids, lipid species, acylcarnitines, and diet-derived metabolites that reflect both dietary exposure and host metabolic status (36). In future rural cancer cohorts, combining diet assessment, nutritional biomarkers, body composition, inflammatory markers, fecal microbiome profiling, and treatment outcome data could clarify how nutritional vulnerability affects tumor subtype, treatment response, recurrence, and survival (Table 3).

**Table 3:** Clinical and epidemiological evidence relevant to nutritional status and cancer outcomes in women.

Evidence category	Main contribution to the review	Relevance to rural Chinese women	Main limitation of current evidence
Dietary-pattern and survivorship studies	Link post-diagnosis diet quality, soy intake, and dietary patterns with recurrence or mortality signals	Supports culturally familiar, diet-quality approaches rather than single-food advice	Rural-specific cohorts remain limited
Preserved-food and high-salt dietary exposure studies	Associate preserved, pickled, and high-salt foods with gastrointestinal cancer risk	Relevant where preserved foods reflect tradition, seasonality, storage limits, or food access constraints	Risk evidence is stronger than post-diagnosis survival evidence
Malnutrition screening studies	Identify patients at risk of complications, poor tolerance, or poorer survival	Feasible if first-line screening is simple, repeated, and embedded in county workflows	Rural implementation and repeat screening remain limited
Nutritional biomarker studies	Use albumin, hemoglobin, lymphocyte count, inflammatory indices, and body composition to stratify risk	May reveal hidden vulnerability before severe weight loss or treatment interruption	Interpretation requires clinical context, including inflammation, infection, liver function, tumor burden, and treatment stage
Body composition and sarcopenia studies	Show that low muscle reserve and sarcopenic obesity may affect tolerance and prognosis	Important where undernutrition-related muscle loss and obesity-related metabolic risk coexist	Routine body composition testing is often unavailable in county-level settings
Metabolomic and microbiome studies	Connect diet, host metabolism, microbial function, inflammation, and treatment response	May clarify mechanisms linking rural dietary exposures with treatment outcomes	Longitudinal rural biospecimen cohorts are sparse, and clinical use requires validation
Implementation and digital-health studies	Test education, symptom monitoring, dietary tracking, referral, and follow-up support	Useful for extending nutrition care beyond tertiary hospitals after discharge	Tools must fit rural literacy, age, infrastructure, staffing, and reimbursement conditions
Health equity and policy studies	Show how insurance, food access, rural service capacity, and referral systems shape supportive care	Frames nutrition as part of rural cancer survivorship equity	Policy studies often do not directly measure nutrition-specific cancer outcomes

## 6 Barriers and health equity challenges in rural cancer nutrition

The translation of nutritional science into improved cancer outcomes is constrained by structural, healthcare-system, community-level, and individual barriers. These barriers accumulate across the cancer care pathway and may place rural women at higher risk of delayed nutritional assessment, inadequate dietary support, poor treatment tolerance, and long-term functional decline.

At the individual and household level, low nutrition literacy may limit a patient’s ability to interpret dietary advice during treatment. Misconceptions about “cancer-promoting foods,” excessive dietary avoidance, or the belief that tumors can be “starved” through severe restriction may worsen iatrogenic malnutrition and delay appropriate nutritional support (37). These problems may be intensified among older women, women in minority regions, and patients with limited access to reliable health information. Nutrition education should therefore include spouses, adult children, and other regular caregivers, rather than placing responsibility for daily nutrition management solely on the patient.

At the healthcare-system level, oncology–nutrition integration remains uneven. County- and township-level facilities may lack dedicated oncology dietitians, routine malnutrition screening, structured referral pathways, and standardized dietary counseling. This creates a mismatch between the clinical need for repeated nutritional assessment and the service capacity available in many rural cancer-care settings. As a result, nutritional decline may not be identified until it has already affected treatment tolerance or functional recovery.

At the structural level, insurance coverage, geographic remoteness, seasonal food supply, and household labor obligations may further restrict nutritional care. Health system reform in China has expanded basic access, but supportive services, rehabilitation, long-term nutrition counseling, and medical nutrition therapy may still be unevenly available across regions (38). For rural women, the burden of agricultural work, household responsibilities, and caregiving may also reduce the time and resources available for follow-up visits, dietary management, and recovery.

These barriers show why rural cancer nutrition should be framed as an equity issue rather than an individual lifestyle issue. Without accessible screening, affordable nutritional support, culturally appropriate counseling, and continuity between tertiary hospitals and local follow-up systems, rural women may remain vulnerable to preventable nutritional deterioration throughout treatment and survivorship.

## 7 Translational opportunities and policy implications

**Figure 4:**
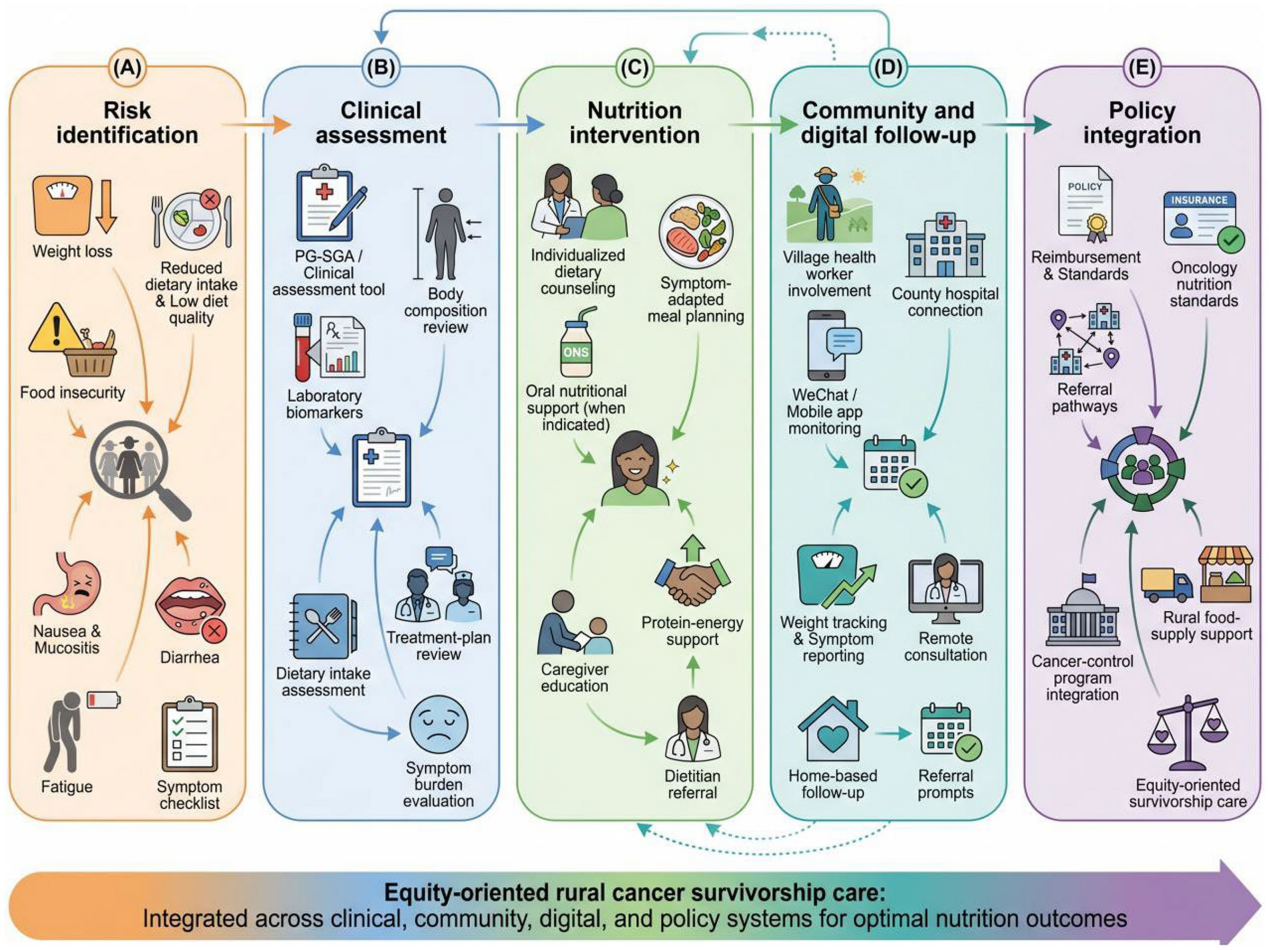
Translational pathway for improving cancer nutrition among rural women. **(A)** Risk identification begins with screening for weight loss, reduced intake, low diet quality, food insecurity, nausea, mucositis, fatigue, and other treatment-related symptoms. **(B)** Clinical assessment integrates structured nutrition tools, body composition indicators, laboratory markers, dietary intake, treatment plans, and symptom burden. **(C)** Nutrition intervention includes individualized dietary counseling, oral nutritional support when indicated, symptom-adapted meal planning, caregiver education, and referral for medical nutrition therapy. **(D)** Community and digital follow-up involve village health workers, county hospitals, mobile symptom reporting, dietary monitoring, remote consultation, and home-based follow-up. **(E)** Policy integration includes reimbursement pathways, county-level nutrition standards, referral systems, rural food-supply support, and integration of nutrition care into cancer-control programs. The pathway emphasizes that rural cancer nutrition requires a connected, equity-oriented system rather than isolated dietary advice.

Improving cancer nutrition among rural women requires a connected strategy that moves from biological rationale to practical implementation. The key task is not simply to tell patients what to eat, but to build a care pathway that identifies nutritional risk early, responds to treatment-related decline, supports patients after discharge, and links clinical nutrition with rural health equity. Figure 4 presents a translational pathway for rural cancer nutrition, moving from risk identification to clinical assessment, intervention, follow-up, and policy integration.

At the research level, prospective rural cancer cohorts should incorporate repeated assessment of diet quality, body composition, nutritional biomarkers, inflammatory markers, metabolomic profiles, and fecal microbiome composition. Large cohort platforms such as the China Kadoorie Biobank demonstrate the value of linking population-level exposure data with long-term disease outcomes (39). Future rural cancer studies could build on this model by adding cancer-treatment variables, nutritional screening, biomarker collection, microbiome profiling, and survivorship follow-up.

At the clinical level, county hospitals and referral networks should distinguish simplified first-line screening from comprehensive assessment. First-line screening can be conducted by nurses or physicians using recent unintentional weight loss, BMI, reduced intake, eating-related symptoms, food insecurity, hemoglobin, serum albumin, total lymphocyte count, and basic functional indicators. Patients who screen positive should receive more comprehensive assessment using the Patient-Generated Subjective Global Assessment (PG-SGA), GLIM criteria, dietary intake review, symptom evaluation, body composition or functional measures when available, and individualized dietary planning (40). This tiered approach is more feasible for county-level oncology care than relying only on comprehensive specialist assessment.

At the community level, task-shifting can extend basic nutrition support beyond tertiary hospitals. Lay health workers and community-based providers have been used in resource-limited settings to deliver structured health education, follow-up, and adherence support (41). In rural cancer care, a similar model could be adapted to provide dietary education, symptom monitoring, weight tracking, referral prompts, and caregiver training that explicitly includes spouses, adult children, and other household decision-makers.

Digital health tools may also help overcome geographic barriers, but they should be designed around rural usability rather than urban assumptions. Mobile health interventions can support dietary tracking, symptom reporting, remote counseling, and patient education when they are simple, culturally adapted, and integrated with clinical workflows (42). For rural women, digital support should not replace local healthcare workers; it should strengthen continuity between provincial oncology centers, county hospitals, village health workers, patients, and caregivers.

At the policy level, nutritional care should be embedded into rural cancer-control strategies. Practical actions include routine malnutrition screening in oncology services, reimbursement pathways for medical nutrition therapy when clinically indicated, referral standards for high-risk patients, and food-system policies that improve access to diverse and affordable nutrient-rich foods. Local agricultural cooperatives, village markets, community food-supply systems, and cold-chain support could be incorporated into survivorship planning to reduce seasonal gaps in fresh food and protein access. Clinical nutrition recommendations support early screening and intervention for cancer-related malnutrition, and health-system evidence from China underscores the need for delivery models that are feasible within rural referral systems (37, 38).

## 8 Conclusion and future directions

Nutritional status is a biologically plausible and clinically meaningful determinant of cancer outcomes among women, yet its role in rural Chinese cancer survivorship remains insufficiently defined. In rural settings, nutritional vulnerability extends beyond undernutrition and reflects the combined effects of dietary transition, low dietary diversity, micronutrient insufficiency, food insecurity, metabolic dysregulation, treatment-related nutritional decline, and limited access to oncology–nutrition services. These factors may influence cancer progression and survivorship through metabolic signaling, adiposity-related inflammation, tumor microenvironment remodeling, gut microbiome-mediated estrogen metabolism, oxidative stress, epigenetic regulation, immune function, and treatment tolerance.

Current evidence supports the need to regard nutrition as an integral component of rural cancer care rather than a peripheral supportive measure. However, most available dietary and survivorship studies are derived from urban or mixed populations, and rural-specific longitudinal evidence remains limited. Mechanistic pathways have been increasingly clarified, but they have rarely been examined in rural Chinese cancer populations with repeated nutritional assessment, biospecimen collection, treatment data, and survival follow-up.

Future studies should establish prospective rural cancer cohorts integrating diet quality, body composition, micronutrient status, inflammatory markers, metabolomics, gut microbiome profiling, treatment tolerance, recurrence, and survival outcomes. Intervention studies are also needed to evaluate whether structured nutritional screening, symptom-adapted dietary counseling, oral nutritional support, caregiver education, and community-based follow-up can improve treatment completion, recovery, quality of life, and long-term prognosis.

From a practice and policy perspective, rural cancer nutrition should be embedded into the broader cancer-control system. County hospitals, township health centers, village clinics, digital follow-up platforms, and tertiary oncology centers should be connected through feasible screening tools, referral thresholds, nutrition-support protocols, caregiver involvement, and reimbursement mechanisms. Such integration may help transform nutrition care from isolated dietary advice into an equity-oriented, mechanism-informed, and implementation-ready strategy for reducing preventable disparities in cancer outcomes among rural Chinese women.
